# Semi-Ownership and Sterilisation of Cats and Dogs in Thailand

**DOI:** 10.3390/ani2040611

**Published:** 2012-11-06

**Authors:** Samia R. Toukhsati, Clive J. C. Phillips, Anthony L. Podberscek, Grahame J. Coleman

**Affiliations:** 1 Animal Welfare Science Center, School of Psychology and Psychiatry, Monash University, Clayton VIC 3800, Australia; E-Mail: grahame.coleman@monash.edu; 2 Centre for Animal Welfare and Ethics, School of Veterinary Science, University of Queensland, Gatton Campus, Gatton QLD 4343, Australia; E-Mail: c.phillips@uq.edu.au; 3 Centre for Animal Welfare and Anthrozoology, Department of Veterinary Medicine, University of Cambridge, Cambridge CB3 0ES, UK; E-Mail: alp18@cam.ac.uk

**Keywords:** sterilise, stray companion animal, semi-ownership, attitudes, beliefs, religion, culture, Thailand

## Abstract

**Simple Summary:**

This study found that less than 15% of Thai nationals engaged in semi-ownership practices, such as feeding, but that few of these stray cats and dogs had been sterilised. Intentions to sterilise in the future were predicted by religious beliefs, attitudes towards sterilisation, perceived pressure from others, and beliefs about personal capacity to sterilise (such as affordability). Community awareness campaigns that approach the issue of sterilisation in a way that is consistent with cultural and religious traditions using Thai role models, such as veterinarians, may go some way in reducing stray animal population growth.

**Abstract:**

The aim of this study was to identify the prevalence of cat and dog semi-ownership in Thailand and factors that predict sterilisation. Semi-ownership was defined as interacting/caring for a companion animal that the respondent does not own, such as a stray cat or dog. A randomised telephone survey recruited 494 Thai nationals residing in Thailand. The findings revealed that 14% of respondents (n = 71) engaged in dog semi-ownership and only 17% of these dogs had been sterilised. Similarly, 11% of respondents (n = 55) engaged in cat semi-ownership and only 7% were known to be sterilised. Using Hierarchical Multiple Regression, the findings showed that 62% and 75% of the variance in intentions to sterilise semi-owned dogs and cats, respectively, was predicted by religious beliefs, and psychosocial factors such as attitudes, perceived pressure from others, and perceived behavioural control. Community awareness campaigns that approach the issue of sterilisation in a way that is consistent with cultural and religious traditions using Thai role models, such as veterinarians, may go some way in reducing stray animal population growth.

## 1. Introduction

Accurate population estimates of stray companion animals in Thailand are not available; however, sources suggest that these number in the millions for cats and dogs [1]. Large, free-roaming populations of stray cats and dogs present substantial risks to the environment [2] and human health, such as the potential for children to be exposed to rabies from dog bites [3]. Stray animals tend to be at least partly dependent on humans for their survival (such as for the provision of food) and people who interact with or care for these animals, but profess not to own them, are termed “semi-owners” [4]. Although communities may benefit from semi-ownership (such as through opportunities for companionship and protection from vermin), semi-owned animals have a high risk of suffering and mortality through inadequate resource provision, untreated illness/disease or injury. Moreover, when semi-ownership comprises feeding, but not sterilising animals, it contributes to the growth of stray populations [4,5] because animals that are sufficiently well fed tend to be able to breed. In Thailand, as in other largely Buddhist nations, the offer of food to stray animals is perceived as a selfless act of kindness and generosity, in keeping with the religious observance of “making merit”. Consideration of cultural and religious traditions will need to feature in community programs that seek to reduce stray animal population growth.

Euthanasia is one of several strategies used to manage populations of unwanted animals in developed nations. In the USA alone, of the six to eight million cats and dogs estimated to enter the shelter system annually, approximately half are euthanized and the remainder rehomed [6]. The efficacy and ethics of this practice to manage stray/feral populations remains the subject of ongoing debate among stakeholders, including wildlife environmentalists, animal advocates, and civil society [7,8,9,10]. Moreover, many cultures are philosophically opposed to this practice. For instance, in keeping with the principle of *ahimsa *(non-injury of all animals) [11], euthanasia does not tend to be practised in Thailand as it contravenes the prevailing Buddhist religion, which requires that humans abstain from destroying living things [1,12]. Alternative methods of humane population management that are culturally acceptable are therefore needed. 

Contraceptives present one such option [10], with single-shot immuno-contraceptives offering a long-lasting method of fertility control in some mammals, including cats [13]. While these hold substantial promise, contraceptives presently in use in Thailand (e.g., medroxyprogesterone acetate), may be associated with serious side-effects, such as pyometra (a dangerous uterine infection), when used long-term [14,15]. Another non-lethal method of managing stray cats and dogs is through Capture-Neuter-Vaccinate-Return (CNVR) programs [10,16,17], although this method also has detractors (see [18] for a summary of the main issues). While there are reports of success [19,20], the capacity for CNVR to reduce, and ultimately eliminate, stray/feral populations remains largely theoretical [21], requires a “long-term commitment of resources” [20] (p. 1361), and may not be appropriate in all situations, particularly those requiring urgent environmental intervention (e.g., when the survival of an indigenous species is threatened by predation from cats). Nonetheless, at the least, sterilisation offers a more ethically palatable alternative to euthanasia and is unlikely to be associated with significant adverse welfare outcomes. 

### 1.1. Animal Population Control in Thailand

Research on community attitudes towards animal population control (APC) interventions in developing nations is scarce. However, this may be particularly pertinent in nations in which (1) large stray companion animal populations are a tolerated feature of community life and (2) cultural/religious traditions discourage interference with natural biological processes, such as breeding. To this end, concerns about sterilisation have previously been raised by Muslim and Buddhist Thais [22,23]. For instance, a Thai interview respondent reported that “If you take them (cats) to a Buddhist vet, they may not even do the desexed thing. I didn’t know that before” [23]. Moreover, we found that most Thai companion animal owners had not sterilised their cat or dog and, of those that had, most believed that sterilisation had not been consistent with their religious beliefs [24]. Other research undertaken in Thailand also confirms low sterilisation rates amongst Thai dog owners [25]. Taken together, these, albeit limited, findings suggest the need for ongoing exploration to determine Thai beliefs, values and attitudes towards sterilisation to achieve APC. 

### 1.2. Psychosocial Determinants of Sterilisation

**Figure 1:**
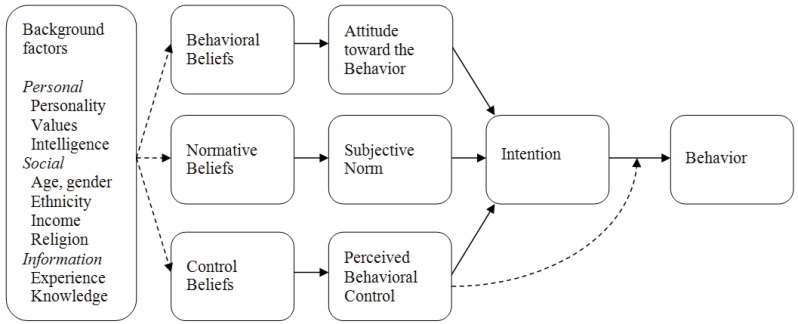
Theory of Planned Behaviour model; Attitudes, personality and behaviour [27].

Stray cats and dogs are said to exist as a function of human attitudes and behaviours, such as semi-ownership practices, however these are poorly understood [26]. Nonetheless, it is generally accepted that attitudes towards a particular behaviour, such as sterilisation, are a key determinant in predicting the enactment of that behaviour. These principles are outlined in the Theory of Planned Behaviour (TPB) which suggests that background demographic and psychosocial factors (such as knowledge, beliefs, and values), influence the formation of attitudes, perceived social norms and perceived personal agency and that these, in turn, influence behaviour (see Figure 1) [27]. For instance, some research has linked personality attributes, such as agreeableness, to attitudes towards animal welfare and their use [28]. Moreover, gender and animal empathy have both been associated with attitudes towards sterilisation practices [29,30,31]. As has been demonstrated previously [32,33], the TPB model offers a means of developing our understanding of attitudes towards companion animal ownership and population control management, such as sterilisation, in non-western cultures. 

Humane APC management is a global challenge that continues to present opportunities for improvement through innovation, partnership and community engagement. While NGOs will no doubt play an integral role in the future of APC management in emerging economies, enhanced community engagement and ownership of such programs is essential for their ongoing sustainability and effectiveness [34]. Greater understanding of local attitudes towards pet ownership practices and APC management is an important way forward in this regard. 

### 1.3. Study Aims

Given that stray populations are at least partly maintained via semi-ownership practices [5], the first aim of this study was to determine the prevalence of semi-ownership of cats and dogs in Thailand. Second, using the TPB, we aimed to identify psychosocial factors that predict intentions to sterilise in semi-owners. 

## 2. Method

### 2.1. Participants and Procedure

A random telephone interview was undertaken between the 11th and 18th March 2011 by Foresight Research Co., Ltd. to recruit 500 Thai nationals aged 18 years or older. The stratified sample comprised equal representation from provinces within Bangkok, Central, Northern, North Eastern and Southern Thailand. Participants were contacted via landline telephone, which are in use by 21.40% of Thai households overall and 50% of households in Bangkok [35]. Potential participants were recruited via stratified, simple random sampling and one attempt was made to contact every 15th or 20th individual listed in the telephone directory. In total, contact was attempted with 5,727 potential participants: no contact was able to be made with 1,683 individuals; 3,544 declined to participate; and 500 agreed. This constituted a 12% recruitment success rate. Six respondents were excluded as non-Thai nationals. The final sample comprised 494 adults (Male = 146, Female = 348), with a mean age of 37.69 years (SD = 12.61), residing throughout rural and non-rural regions in Thailand. Of these, 102 respondents (20.65%) indicated that they regularly interact with and care for a cat and/or dog that they do not claim to own and were thus deemed “semi-owners”. This sub-sample (n = 102) comprised 67 females and 35 males with a mean age of 38.11 years (SD = 13.21). Most were technically/tertiary educated (66.7%), employed full time (61.8%), Buddhist (96.1%), and married (57.8%) with an average monthly income of 13000THB (approximately equivalent to $410USD). The subsample of semi-owners (n = 102) was similar to the larger sample of non-semi-owners (n = 392) in terms of age, gender distribution, region of residence, education, employment, religion, relationship status and monthly income, with no significant differences between groups identified (*p* > 0.05). Moreover, these characteristics are broadly in keeping with national Thai statistics [35], with the exception that this cohort were somewhat more highly educated and had greater access to landline telephones (as was the also case for the total sample, N = 494). 

### 2.2. Materials

The Thai version of the Culture and Human-Animal Interactions (CHAI) survey was used to interview respondents in relation to:

(1) Demographic characteristics: gender, age, highest education attained (e.g., Primary, Secondary, Tertiary), residency (e.g., Urban, Suburban, Rural), employment status (e.g., Full Time, Part Time), relationship status (e.g., Single, Married, Divorced/Widowed), religion (e.g., Buddhist, Muslim), spoken language, income, pet keeping and sterilisation practices.
(2) Psychosocial (TPB) factors: knowledge (e.g., “Female cats and dogs need to have one litter before being desexed”), beliefs (e.g., “De-sexing this cat is consistent with my religious beliefs” and “Sterilization makes cats overweight”), subjective norms (e.g., “People who are important to me think that I should de-sex this cat”), perceived behavioural control (e.g., “Financial constraints prevent me from de-sexing this cat”) and intentions (e.g., “I intend to de-sex this cat in the near future”) regarding sterilisation. Items were answered using a five point Likert-type scale, anchored by 1 = strongly disagree and 5 = strongly agree. Attitudes towards sterilisation were indexed using opposing attitudinal descriptors (e.g., harmful *vs.* beneficial) [36]; higher scores represent more positive attitudes towards sterilisation.
(3) Animal Empathy Scale [37]: eight positively and eight negatively phrased empathy items indexed using a five point Likert-type scale (1 = strongly disagree and 5 = strongly agree). Total scores could range from 16 to 80 and high scores indicated higher animal empathy.
(4) The Ten Item Personality Inventory [38]: provided an index of personality characteristics using a 5 point, Likert-type scale, where 1 = strongly disagree and 5 = strongly agree. Five personality dimensions were examined including extraversion, agreeableness, conscientiousness, emotional stability and openness to new experiences. High scores indicate a tendency towards the specified trait.

### 2.3. Statistics

Principle Components Analyses was used to identify constructs drawn from multiple items; subscales with acceptable internal consistency (Cronbach’s alpha ≥ 0.7) were used in subsequent analyses. Correlations and Hierarchical Multiple Regression (HMR) models were used to examine demographic and psychosocial factors that predict intentions to sterilise semi-owned cats and dogs in the near future (1 = strongly disagree and 5 = strongly agree). Cook’s distance (<1) confirmed the normality of the residuals. The structure of the HMR Model can be seen in Table 1 (categorical demographic variables were dummy coded). Alpha was set at *p* ≤ 0.05.

**Table 1:** Variables in the Hierarchical Multiple Regression Model.

Step	Variable Type	*Variables*
**1**	**Demographics**	*Region in Thailand*
		*Gender*
		*Age*
		*Education*
		*Employment status*
		*Religion*
		*Relationship status*
		*Residential location*
		*Monthly income*
**2**	**Personality**	*Extraversion*
		*Agreeableness*
		*Conscientiousness*
		*Emotional Stability*
		*Openness*
**3**	**Empathy**	*Animal Empathy*
**4**	**Beliefs**	*Cats and dogs are independent*
		*Education about cat and dog interactions is important*
**3**	**Desexing outcome beliefs**	*Negative sterilisation outcomes *
**4**	**Theory of Planned Behaviour **	*Attitudes towards desexing*
		*Norms—Desexing is endorsed by important others*
		*Religion—Desexing is consistent with my religious beliefs*
		*PBC ^1^—Agency (Desexing was/is entirely my decision)*
		*PBC—Arrange (I could arrange desexing if I wanted to) *
		*PBC—Access (Limited access to veterinarians near my home prevents me from desexing) *
		*PBC—Finances (Financial constraints prevent me from de-sexing this dog/cat )*
		*PBC—Commitments (My commitments prevent me from desexing )*

^1^ PBC = Perceived Behavioural Control

## 3. Results

### 3.1. Cat Semi-Ownership and Sterilisation

Of the entire sample (N = 494), 55 respondents (11.13%), comprising 18 males (32.7%) and 37 females (67.3%), indicated that they regularly interact with and care for a cat they do not own. Of these semi-owned cats, there were somewhat more females relative to males (52.7% *vs.* 47.3% respectively) and 36.4% wore collars. Most cat semi-owners did not think that they were the main person who looked after the cat (76.4%). In relation to ownership, 45.5% believed the cat was owned by someone, 30.9% believed the cat was unowned, and 23.6% were unsure. Only 7.3% the cats were known to be desexed (n = 4), with 60% known to be intact and 32.7% of semi-owners were unsure. 

### 3.2. Dog Semi-Ownership and Sterilisation

In relation to semi-owned dogs, 71 respondents, comprising 23 males (32.4%) and 48 females (67.6%), indicated that they interact with and care for a dog they do not own; this represented 14.37% of the entire sample (N = 494). Most of the semi-owned dogs were male (59.2%) and did not wear collars (74.6%). Almost half of the dog semi-owners (49.3%) believed the dog was owned by someone else, 33.8% thought the dog was unowned and 16.9% were unsure. Most dog semi-owners did not believe that they were the main person looking after the dog (76.1%). Only 16.9% of semi-owners thought the dog had been desexed (n = 12), 66.2% indicated that the dog was intact and a further 16.9% were unsure. 

Of the sample of semi-owners (n = 102), most (n = 73, 71.6%) also indicated ownership of a cat and/or a dog.

### 3.3. Religion and Sterilisation

**Figure 2:**
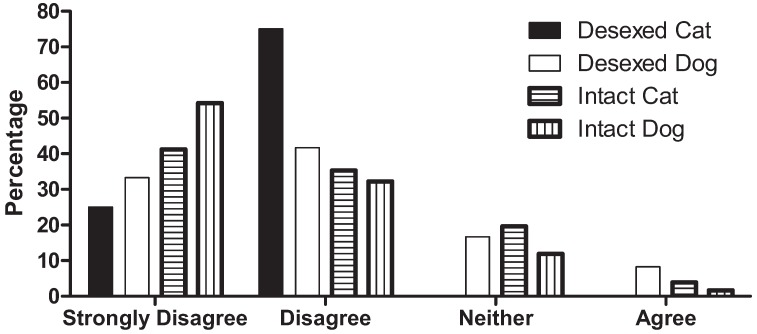
Percentage agreement that sterilisation was/is consistent with religious beliefs in semi-owners of intact or sterilised cats and dogs.

Mean agreement that sterilisation had been consistent with respondents’ religious beliefs was low for dog semi-owners (n = 12, Mean = 2.00, SD = 0.95) and cat semi-owners (n = 4, Mean = 1.75, SD = 0.50). Similar findings were observed for semi-owners of intact animals, with most indicating disagreement that the decision to sterilise their intact semi-owned cat (n = 51, Mean = 1.86, SD = 0.87) or dog (n = 59, Mean = 1.61, SD = 0.77) would be consistent with their religious beliefs (see Figure 2). 

### 3.4. Attitudes towards Sterilisation

**Figure 3:**
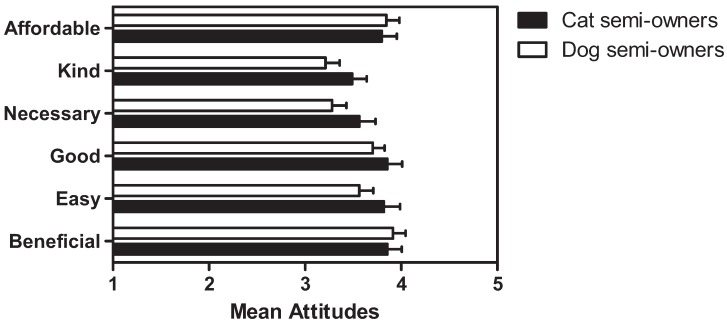
Mean attitudes (±SEM) towards sterilisation in cat and dog semi-owners (1 = strongly disagree, 5 = strongly agree).

Semi-owners of cats (n = 55) and dogs (n = 71) were asked to indicate their attitudes towards sterilisation. As can be seen in Figure 3, attitudes towards sterilisation among cat and dog semi-owners were moderately positive and no significant differences were observed between groups. In addition, no significant differences in attitudes towards sterilisation as a function of semi-owner gender emerged. 

### 3.5. Beliefs Regarding Health and Behavioural Outcomes of Sterilisation

**Figure 4:**
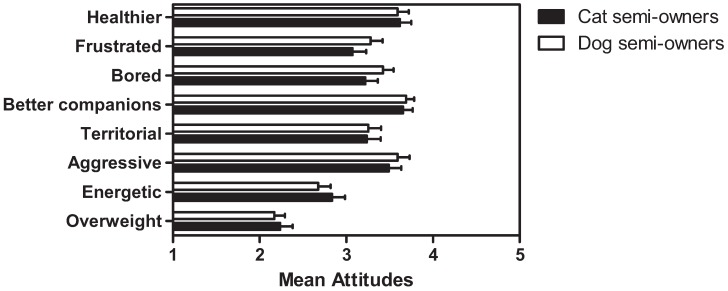
Mean beliefs (± SEM) regarding the outcomes of sterilisation in cat (n = 55) and dog (n = 71) semi-owners (1 = strongly disagree, 5 = strongly agree).

Cat (n = 55) and dog (n = 71) semi-owners were asked their beliefs about the health and behavioural outcomes of sterilisation for cats and dogs. As shown in Figure 4, the findings indicated some positive (*i.e.*, health, companionship) and some negative (*i.e.*, frustrated, bored, territorial, aggressive) beliefs about sterilisation outcomes. Semi-owners believed that sterilised animals become less energetic, but not overweight. There were no significant differences between cat and dog semi-owners.

### 3.6. Knowledge and Beliefs about Pet Care Practices

In general, semi-owners (n = 102) and non-semi-owners (n = 392) demonstrated moderate knowledge regarding pet care practices. Few significant differences between these groups emerged (see Table 2). 

**Table 2:** Mean agreement with knowledge and belief statements regarding companion animal issues by semi-ownership category (1 = strongly disagree, 5 = strongly agree).

Knowledge and Belief Items	Semi- Owner (n = 102)		Non- Semi-Owner (n = 392)		*p*
	Mean	SD	Mean	SD	
Female cats and dogs need to have one litter before being desexed	2.87	1.35	2.69	1.13	*p* > 0.05
Food prepared for humans is suitable for cats and dogs	2.88	1.25	2.65	1.10	*p* > 0.05
The needs of cats and dogs in Asia are different from those in the West	2.37	0.98	2.51	0.93	*p* > 0.05
Dog bites are a serious problem in the community	4.25	0.92	4.18	0.78	*p* > 0.05
Desexing male cats and dogs feminises them	3.19	1.08	3.05	0.95	*p* > 0.05
It is not a waste of money to pay for cat desexing	3.67	1.18	3.62	0.87	*p* > 0.05
Desexing deprives cats and dogs of the right to sexual activity	3.49	1.15	3.45	0.94	*p* > 0.05
Cats are lovable family members	3.98	0.95	3.86	0.85	*p* > 0.05
Dogs are lovable family members	4.35	0.61	4.20	0.58	***p*** ** < 0.05**
Cats can take care of themselves	3.70	1.18	3.64	0.97	*p* > 0.05
Dogs can take care of themselves	3.50	1.17	3.39	1.07	*p* > 0.05
Children should learn how to interact with cats	4.18	0.70	4.00	0.85	***p*** ** < 0.05**
Children should learn how to interact with dogs	4.25	0.66	4. 06	0.75	***p*** ** < 0.05**
Dogs can scavenge adequate food for themselves	2.79	1.15	3.08	1.07	***p*** ** < 0.05**
Cats can scavenge adequate food for themselves	3.28	1.13	3.19	1.09	*p* > 0.05

### 3.7. Agency, Norms and Sterilisation

Mean agreement regarding normative pressure and perceived agency to sterilise was examined in cat and dog semi-owners. In general, all respondent groups indicated low to moderate agreement that important others would support sterilisation or that the decision to sterilise had been entirely up to them (see Table 3). Semi-owners of intact semi-owned cats and dogs further indicated low to moderate agreement that perceived obstacles prevented sterilisation (Table 4). 

**Table 3:** Mean agreement with agency and norm statements by semi-ownership category (1 = strongly disagree, 5 = strongly agree).

Agency, Norms	Semi-owner of sterilised cat (n = 4)		Semi-owner of sterilised dog (n = 12)		Semi-owner of intact cat (n = 51)		Semi-owner of intact dog (n = 59)	
	Mean	SD	Mean	SD	Mean	SD	Mean	SD
The decision to sterilise was/is entirely up to me	1.75	0.50	2.75	1.48	2.22	1.21	2.10	1.18
People who are important to me think that I should sterilise	2.25	0.96	2.67	1.56	2.27	1.04	2.17	1.10

**Table 4:** Mean agreement with perceived behavioural control statements by semi-owners of intact cats and dogs (1 = strongly disagree, 5 = strongly agree).

Perceived Behavioural Control	Semi-owner of intact cat (n = 51)		Semi-owner of intact dog (n = 59)	
	Mean	SD	Mean	SD
Limited access to veterinarians near my home prevents me from sterilising	2.25	1.00	2.00	0.95
Financial constraints prevent me from sterilising	2.25	1.18	2.08	1.16
My commitments prevent me from sterilising	2.16	1.05	2.14	1.18
I could arrange to have this cat/dog sterilised if I wanted to	2.69	1.24	2.44	1.30

### 3.8. Animal Empathy Scale

Total mean animal empathy scores in semi-owners (n = 102) ranged from 45 to 76.9, with a mean of 58.13 (SD = 8.05), indicating a tendency towards animal empathy. Animal empathy was significantly higher in semi-owners compared with non-semi-owners (n = 392, M = 54.53, SD = 5.87), *t*(492) = 5.07, *p* < 0.001. Within the sample of semi-owners (n = 102), cat semi-owners (n = 31, M = 55.03, SD = 6.62) had less empathy towards animals than dog semi-owners (n = 47, M = 59.27, SD = 7.87) or cat/dog semi-owners (n = 24, M = 59.87, SD = 9.20), F(2,101) = 3.50, *p* < 0.05.

### 3.9. Personality, Attitudes and Empathy

The TIPI was used to examine personality characteristics in semi-owner (n = 102) *vs.* non-semi-owner (n = 392) groups. Mean scores for both groups were around the midway point of the 5-point scale for each of the personality domains (extraverted, agreeable, conscientious, emotional stability and openness). The results showed that semi-owners were significantly more open to experience than non-semi-owners, *t*(492) = 3.46, *p* < 0.001. Pearsons correlation coefficients were used to identify associations between personality, attitudes and empathy in semi-owners (n = 102) (see Table 5). With the exception of extraversion, all personality domains were associated with attitudes towards desexing and animal empathy. 

**Table 5:** Pearson’s Correlation Coefficient between personality, attitudes and empathy in semi-owners (n = 102).

	Extravert	Agreeable	Conscientious	Emotional Stability	Openness
Attitudes towards desexing dogs	0.07	0.12 *	0.15 **	0.06	0.12 **
Attitudes towards desexing cats	0.01	0.12 **	0.13 **	0.11 *	0.12 **
Animal empathy	−0.08	0.17 **	0.13 **	−0.11 *	0.17 **

*p < 0.05; ** *p* < 0.001

### 3.10. Intentions to Desex Semi-Owned Cats and Dogs in the Future

The findings revealed low intentions for cat (Mean = 2.12, SD = 1.14) and dog (Mean = 1.17, SD = 0.99) semi-owners to sterilise in the near future; differences between groups were not significant. In total, 66.7% of cat semi-owners and 79.7% of dog semi-owners strongly disagreed or disagreed with the statement “I intend to desex this cat/dog in the near future”. 

Point biserial correlations failed to identify any associations between demographic factors and intentions to sterilise semi-owned cats and dogs in the near future (*p* > 0.05). There was an inverse association between intentions to desex semi-owned dogs and agreeableness (*r* = −0.37, *n* = 59, *p* < 0.01) and conscientiousness (*r* = −0.26, *n* = 59, *p* < 0.05), but apart from these, personality and animal empathy were not associated with intentions to sterilise semi-owned animals in the future (Table 6). In general, intentions to sterilise semi-owned cats and dogs were associated with TPB variables including attitudes, religious beliefs, norms and perceived behavioural control variables (e.g., agency, financial constraints, access to veterinarians, commitments). 

**Table 6:** Point Biserial Correlation Coefficient between intentions to sterilise and psychosocial factors.

Factor		Intentions to sterilise	
		Semi-owned Dog (n = 59)	Semi-owned Cat (n = 51)
**Personality**	Extraversion	0.13	−0.20
	Agreeable	−0.37 **	−0.11
	Conscientious	−0.26 *	−0.03
	Emotional Stability	−0.17	0.09
	Openness	−0.10	−0.11
**Animal empathy**		−0.02	−0.19
**Beliefs**	Independent	−0.13	−0.29 *
	Education	−0.21	−0.04
**Desexing outcome beliefs**		0.08	−0.02
**Theory of Planned Behaviour**	Attitude: positive	0.30 *	0.22
	Norms	0.66 **	0.43 **
	Religion	0.55 **	0.54 **
	PBC ^1^ – agency	0.54 **	0.61 **
	PBC – access	0.53 **	0.50 **
	PBC – finances	0.36 **	0.36 **
	PBC – commitments	0.44 **	0.50 **
	PBC – arrange	0.49 **	0.37 **

^1 ^PBC = Perceived Behavioural Control; * *p* < 0.05; ** *p* < 0.001

The HMR model for intentions to sterilise semi-owned dogs in the future was highly significant F(9, 48) = 11.45, *p* < 0.001. Respondents living in Southern Thailand (*β* = −0.31) and those that were agreeable (*β* = −0.26) were less likely to hold intentions to sterilise semi-owned dogs in the future. Conversely, respondents who perceived normative pressure to sterilise (*β* = 0.46), perceived sterilizing to be consistent with his/her religion (*β* = 0.40), considered the decision to sterilise entirely theirs to make (*β* = 0.42), believed that sterilisation was affordable (*β* = 0.21), but that financial constraints prevented them from sterilising (*β* = 0.27) and did not believe that they could make arrangements to sterilise if they wanted to (*β* = −0.48), were more likely to hold intentions to sterilise. These factors accounted for 62.3% of the variance in intentions to sterilise semi-owned dogs in the future (see Table 7). 

A significant HRM model was also achieved for intentions to sterilise semi-owned cats in the future, F(7, 42) = 21.59, *p* < 0.001. Respondents who had only attained a Primary school level of education (*β* = −0.32) and that held the belief that they could not arrange to have the cat desexed if they wanted to (*β* = −0.26) were less likely to intend to sterilise semi-owned cats in the near future. Conversely, respondents who perceived normative pressure to sterilise (*β* = 0.28), believed that the behaviour was consistent with their religious beliefs (*β* = 0.20), believed that the decision to desex was entirely theirs to make (*β* = 0.62), and that personal commitments prevent desexing (*β* = 0.36) were more likely to intend to sterilise semi-owned cats in the future. These factors explained 74.6% of the variance in intentions to sterilise semi-owned cats in the near future (Table 7). 

**Table 7:** Hierarchical Multiple Regression Model for factors predicting intentions to sterilize in cat and dog owners and semi-owners.

	Predictors	b	SE	*β*	*t*	*Adjusted R^2^*
**Semi-owned dog**	South	−0.94	0.29	−0.31 **	−3.21	0.62
	Agreeable	−0.33	0.13	−0.26 *	−2.65	
	Norms	0.44	0.14	0.46 **	3.04	
	Religion	0.51	0.16	0.40 **	3.12	
	PBC ^1^ -decision	0.35	0.11	0.42 **	3.14	
	PBC-finances	0.23	0.10	0.27 *	2.41	
	PBC-arrange	−0.37	0.14	−0.48 *	2.67	
	Attitude-affordable	0.17	0.08	0.21 *	2.08	
**Semi-owned cat**	Education-Primary	−0.92	0.23	−0.32 **	−3.93	0.75
	Norms	0.30	0.11	0.28 *	2.90	
	Religion	0.26	0.12	0.20 *	2.31	
	PBC-decision	0.59	0.10	0.62 **	5.92	
	PBC-commitments	0.40	0.09	0.36 **	4.42	
	PBC-arrange	−0.24	0.10	−0.26 *	−2.32	

^1 ^PBC = Perceived Behavioural Control; * *p* < 0.05; ** *p* < 0.001

## 4. Discussion

This study sought to determine the prevalence of, and attitudes toward, cat and dog semi-ownership and sterilisation behaviours in Thailand. The small proportion of the total sample (N = 494) that indicated semi-ownership of a dog (14%) and/or a cat (11%) appears lower than that observed in Australia, where it has been reported that 22% of Victorians engaged in cat semi-ownership behaviours [4]. Of the 102 semi-owners, most (n = 73) combined cat and/or dog ownership with semi-ownership, which has also been reported in Australia [4]. The comparatively low level of semi-ownership in Thailand may reflect some difficulty in meeting the financial burden of caring for “unowned” animals in addition to those already owned. Research that explores community perceptions of, and attitudes towards, a broader suite of behaviours involved in pet ownership, and semi-ownership, is needed to understand this further; qualitative research is presently underway in Thailand to this effect. 

The rates of sterilisation of semi-owned cats and dogs were low (7.3% and 16.9% respectively) and semi-owners of intact animals indicated low intentions to sterilise, with most cat and dog semi-owners (66.7% and 79.7% respectively) strongly disagreeing or disagreeing that this was something they intended to do in the near future. Given a cultural context in which the tradition of “making merit” through feeding those less fortunate is well entrenched, it is likely that this population of semi-owned cats and dogs are sufficiently well nourished to breed. Further research that specifies semi-owner feeding behaviours, knowledge of breeding habits among semi-owned animals, and beliefs regarding the practice of making merit through feeding will provide greater clarity in this regard.

### 4.1. Psychosocial Factors and Intentions to Sterilise in The Future

In keeping with the TPB [27], we sought to explore the extent to which background (*i.e.*, demographic, personality, religious and knowledge factors) and psychosocial (such as attitudes and perceived norms) factors predicted intentions to sterilise semi-owned cats and dogs in the future. The results revealed strong support for the TPB model, such that 62% and 70% of the variance in intentions to sterilise cats and dogs in the near future, respectively, was explained by attitudes, perceived norms and perceived behavioural control variables. Unlike some past research [30] gender was generally unrelated to attitudes towards sterilisation practices. The findings generally suggest that positive attitudes towards sterilisation and the perception that important others would endorse this practice predicted a greater likelihood of sterilizing in the future; community awareness programs that promote the benefits of sterilizing and normalize it through endorsement by Thai opinion leaders may encourage future engagement in this practice. Towards this end, qualitative research with Thai veterinarians is underway to understand their role in shaping community pet keeping behaviours. 

Several PBC factors were also related to sterilisation intentions in semi-owners. Perceived personal agency with regard to the decision to sterilise was among the most influential predictors; community awareness programs that encourage responsible ownership, in keeping with Thailand’s merit-making traditions, may go some way towards resolving this issue. Additional PBC factors, such as the perceived capacity to make arrangements to sterilise, access to veterinarians, and personal and financial commitments, also predicted intentions to sterilise in the future. Taken together, these findings suggest several perceived barriers to desexing, which may explain why those intending to sterilise have not already done so. The prominence of financial and access obstacles suggests the potential utility of mobile, low cost sterilisation programs that have proven successful elsewhere [39]. 

Our findings further revealed that religious beliefs negatively intersected sterilisation intentions such that 75% or more semi-owners believed that this practice would not be consistent with their religious beliefs. Moreover, of the small number of semi-owners who had already sterilised their animals, the majority (≥75%) did not believe that this had been consistent with their religious beliefs. It can be speculated that, in a largely Buddhist nation, animal population control strategies that demonstrate their alignment with religious traditions may be more successful, for example, by “preventing the future suffering of cats that are without homes and being destroyed by the authorities” [40]. Community consultation is needed in this regard to develop strategies that optimise this approach. 

### 4.2. Limitations

In light of the low recruitment success rate, it is possible that the findings presented here may not be representative of Thai nationals in general. The extent to which a response bias (e.g., such as affluence) and/or an under-coverage bias (e.g., the use of landline telephones for recruitment) may impact knowledge of, and capacity/motivation to engage with, APC strategies remains unknown. Future research that includes affluence in stratification and that uses randomly generated mobile phone numbers may go some way towards addressing these issues.

## 5. Conclusions

In the absence of effective partnerships between government and non-government organizations [3], the long-term sustainability of APC programs dictates even greater need for community initiation, co-operation, and engagement [34]. However, community engagement in such programs is often minimal [22]. While situational factors account for some of the low participation rates, our research has shown that psychosocial factors are an important component of intentions to engage in sterilisation practices. 

Our research provides new insights regarding the knowledge base, attitudes, perceived barriers and sterilisation practices of Thai semi-owners that may be used to guide non-government APC initiatives. In this regard, responsible ownership education programs that emphasise their alignment with cultural/religious beliefs and values (*i.e.*, concern for the well-being of animals) may better engage communities. The development of any such programs should engage communities through consultation to engender inclusive and sustainable methods that address the needs of all stakeholders. Community development APC strategies that emphasise their alignment with cultural/religious traditions and that promote personal agency, access, and affordability using Thai role models are likely to have greater, long-lasting success. 
